# Prosocial, aggressive, or both? A multilevel latent profile analysis of peer status and social behavior in early adolescence

**DOI:** 10.1111/jora.70149

**Published:** 2026-01-26

**Authors:** Katja Košir, Tina Pivec

**Affiliations:** ^1^ Department of Psychology, Faculty of Arts University of Maribor Maribor Slovenia; ^2^ Educational Research Institute Ljubljana Slovenia

**Keywords:** bullying, latent profile analysis, likability, multilevel approach, popularity, prosocial behavior

## Abstract

Adolescence is crucial for shaping social behavior, with peers influencing popularity and likability. While some adolescents use bullying to gain popularity, prosocial behavior often underlies likability. Yet, little is known about bistrategic control, where youth combine aggression and prosocial actions. This study used a multilevel person‐centered approach to identify latent profiles based on peer‐reported bullying, prosocial behavior, popularity, and likability, examined differences in bystander behavior, social status goals, and insecurity, and further explored how these individual‐level profiles differ across classrooms and align with classroom‐level bullying and prosocial social status norms. Data from 6379 Slovenian adolescents in 328 classes revealed five profiles: Unpopular bullies, Popular bullies, Bistrategic, Prosocial, and Uninvolved. At the classroom level, two distinct profiles emerged, differing in the prevalence of Uninvolved, Prosocial, Bistrategic, and Popular bully students.

## INTRODUCTION

Adolescence is a critical period for developing social behavior, with peer interactions shaping social status and behavior. Social status is commonly conceptualized in terms of popularity (social visibility and influence) and likability (peer acceptance) (Cillessen, 2009, 2011). Adolescents may pursue these goals through different means: those focused on popularity often use aggression and bullying, while those seeking likability favor prosocial behaviors (Dawes, 2017; Li & Wright, 2014). Some youth, however, combine both strategies—a pattern known as *bistrategic control*—which remains less understood due to inconclusive empirical findings (Berger et al., 2015; Hartl et al., 2020). Understanding these individuals is crucial, as their social centrality and ability to both cooperate and coerce may shape peer relations and the classroom climate (Hawley, 1999, 2014). To address the gaps regarding the strategies adolescents use to achieve their social status, the present study examined the existence, prevalence, and psychosocial correlates of bistrategic youth by identifying student profiles based on peer‐reported popularity, likability, bullying, and prosocial behavior. Furthermore, the differences among the profiles in terms of self‐reported bystander behaviors in bullying situations, social goals, and social status insecurity will be examined. This person‐centered, multisource design offers a comprehensive view of adolescent social dynamics, combining large‐scale peer data with self‐reports to cross‐validate identified profiles.

Classrooms differ in the extent to which prosocial or bullying behaviors are linked to popularity, shaping how students express their social behaviors (Laninga‐Wijnen & Veenstra, 2021). These classroom social status norms reflect the extent to which certain behaviors, such as bullying and prosocial behavior, are associated with being popular or rejected within a specific classroom context. In classrooms where bullying is rewarded with popularity, students may engage in more bullying, while strong prosocial norms encourage cooperative behavior (Dijkstra & Gest, 2015). Conversely, when bullies are disliked or prosocial students are less disliked, less bullying tends to occur (Garandeau et al., 2022). Such social status norms influence both individual behavior and group dynamics, affecting how defending or reinforcing bullying impacts social status (Garandeau et al., 2022; Peets et al., 2015). To capture these contextual effects, this study applies a multilevel person‐centered approach to explore classroom‐level configurations based on the composition of individual behavior status profiles and to examine how these class profiles differ in four types of social status norms (bullying/prosocial popularity, bullying/prosocial rejection).

### Social status and social behavior: Behavioral and social status profiles among early adolescents

Adolescence is a developmental stage marked by an increased focus on social status (LaFontana & Cillessen, 2010). During this period, adolescents often strive for two types of peer approval: peer acceptance or likability, where they aim to fit in and be liked, and social recognition or popularity, where they seek to stand out, gain admiration, or exert influence within their peer group (Cillessen, 2011; Dawes & Xie, 2014). While these constructs are distinct, they can overlap, as some adolescents may be both well‐known and well‐liked, whereas others may be only popular or likable, or neither (van den Berg et al., 2020).

Behaviors that are rewarded with higher social status can be seen as strategic tools to gain or maintain a high position in the peer group, shaping norms on how to fit in and avoid being a social misfit (Wright et al., 1986). Since popularity is a limited resource requiring constant effort (Hawley, 1999), those prioritizing it may feel pressure to protect or improve their standing. According to Resource Control Theory (Hawley, 1999), both prosocial and aggressive behaviors can serve as means to obtain or maintain social and material resources. Thus, students may use both forms of behavior strategically to achieve social goals (Lansu & van den Berg, 2024). Those who effectively employ both strategies, referred to as “bistrategic controllers” (Hawley, 2014), may use bullying in interactions with some peers while engaging in prosocial behaviors with others (Closson, 2009; Hawley, 2003; Hawley & Bower, 2018). This bistrategic behavior has been linked to greater popularity (Closson, 2009; Lansu, 2023) and possibly to enhanced social skills that offset the negative impact of aggression (Hawley, 2007). To clarify how these various behavioral strategies relate to adolescents' psychosocial adjustment, previous research has sought to identify distinct profiles of adolescents with varying social status and social behavior configurations. Hawley's (2003) seminal classification into coercive, prosocial, bistrategic, typical, and noncontroller groups provided an early conceptual foundation, though based on cut‐off methods. Prosocial and bistrategic controllers were found to be seen as popular and socially accepted, with bistrategic controllers having the highest resource control. Coercive controllers followed in terms of resource control, while typicals and noncontrollers scored lower in peer acceptance and teacher evaluations.

Similarly, other researchers (e.g., Peeters et al., 2010; Reijntjes et al., 2018; Rodkin et al., 2000) have categorized students based on their bullying and prosocial behaviors, though these studies often failed to consider students' social status in conjunction with their behavior. Moreover, many of these studies relied on variable‐centered analytical approaches, focusing on relationships between constructs rather than on identifying distinct combinations of behaviors and status among individuals. More recent research has therefore adopted person‐centered methods, such as latent class or latent profile analysis, to classify participants in bullying and social status research (e.g., Kochel et al., 2015; Stefanek et al., 2017; Yang et al., 2016). However, only a few studies have examined both students' social behavior and social status simultaneously. Berger et al. (2015) used latent profile analysis to classify early adolescents based on aggressive and prosocial behavior, social status, machiavellianism, and empathy, identifying three profiles: normative low aggressive, high prosocial‐low aggressive, and high aggressive‐high popular. They found no profile combining both aggressive and prosocial behaviors (i.e., bistrategic controllers). In contrast, Hartl et al. (2020) identified distinct profiles, including bistrategic, aggressive, and prosocial popularity profiles, with bistrategic adolescents being the most popular and showing high levels of both aggression and prosocial behavior. Similarly, Clark et al. (2020), using self‐reports on resource control strategies, identified a bistrategic controller profile as one of the resource control profiles (others were noncontroller, prosocial controller, low typical controller, and moderate typical controller); however, the purely coercive resource control group was absent in their study.

Together, these findings provide mixed evidence regarding the existence and characteristics of adolescents who combine prosocial and aggressive strategies to attain social status. Given that bistrategic behavior is conceptualized not merely as a behavioral characteristic but as goal‐directed and strategic in nature, it is important to consider adolescents' social motivations when examining this profile. Goal theory (Austin & Vancouver, 1996) suggests that a student's current popularity and likability may reflect an initial intention or goal related to social standing. Such goals might involve becoming popular or likable, as well as maintaining or enhancing their existing social status (Ojanen & Findley‐Van Nostrand, 2014). Popularity goals reflect aspirations to increase one's social visibility and power within the peer group (Dawes, 2017; Kiefer & Wang, 2016), whereas likability goals emphasize being well‐liked and accepted. These goals have been linked to distinct behavioral tendencies: students with strong popularity goals are more likely to engage in bullying and social aggression (e.g., Caravita & Cillessen, 2012; Li & Wright, 2014; van den Broek et al., 2016), whereas those with likability goals tend to employ prosocial behaviors that enhance peer acceptance (Closson, 2009; Lansu, 2023). Moreover, high likability goals have been associated with lower levels of relational aggression (Li & Wright, 2014) and unrelated to bullying behavior (Garandeau & Lansu, 2019). Finally, social status insecurity—the concern about (losing) one's status—has been linked to both overt and relational aggression (Li et al., 2010; Li & Wright, 2014), with students who have high popularity goals and high insecurity reporting higher levels of bullying as a way to manage their social standing (Košir et al., 2022). Thus, incorporating students' social goals and perceptions of status can clarify the motivational basis of the bistrategic profile and link behavioral patterns with underlying intentions.

### Reinforcing bullying and defending as social strategies

While individual goals and motivations are important in shaping social behavior, classroom context plays a critical role in enabling or constraining certain behavioral strategies. Bullying, in particular, is a group‐based phenomenon that is shaped by peer dynamics and social norms. It involves the repeated and intentional abuse of power by a dominant individual or group toward a less powerful peer (Olweus, 1999) and can also be viewed as a strategic behavior aimed at securing social resources within the peer group (Volk et al., 2017). According to the socioecological perspective (Bronfenbrenner, 1979; Espelage & Swearer, 2010), bullying occurs within broader social systems, where peers play a central role as reinforcers or defenders (Salmivalli & Voeten, 2004). Reinforcers indirectly support bullying by providing social rewards—such as laughing or cheering—which can enhance the bully's visibility (Salmivalli, 2010). Students who reinforce bullying often have high peer status, being disliked individually but seen as popular (Pouwels et al., 2016). In adolescence, bullies and their supporters tend to be more popular than defenders (Pouwels et al., 2018).

In contrast, defending—helping or supporting victims—represents a prosocial form of status‐seeking. Defenders may gain visibility and respect by challenging bullies, thereby improving both popularity and likability (Meter & Card, 2015; Salmivalli et al., 2011; Veenstra et al., 2013). Although defending can be perceived as socially risky (Pöyhönen et al., 2010), recent evidence suggests it does not harm social standing and may even strengthen it (Laninga‐Wijnen et al., 2023; Lubon et al., 2024; van der Ploeg et al., 2017). Thus, defending the victim is not only a safe behavior but can also serve as a strategy to increase social status.

### The role of classroom social status norms in students' social behavior

The classroom serves as a crucial social context where students negotiate peer relationships (Eccles & Roeser, 2011). Classroom norms—shared beliefs about acceptable behavior—guide students' actions through social rewards or sanctions (Laursen & Veenstra, 2021). These norms are often shaped by influential peers whose behavior defines the popularity norm (Dijkstra et al., 2008; Veenstra & Lodder, 2022). Popularity norms indicate which behaviors are associated with high social status and are typically operationalized as the within‐classroom correlation between a specific behavior and perceived popularity (Laninga‐Wijnen & Veenstra, 2021). Two types of popularity norms are frequently studied: prosocial and aggressive, which can coexist within the same classroom (Laninga‐Wijnen, Steglich, et al., 2020). Classrooms with stronger aggressive popularity norms tend to show more peer rejection, higher victimization, and poorer adjustment (Dijkstra & Gest, 2015).

The development of classroom popularity norms depends on classroom composition and group dynamics. In classrooms with fewer popular and more unpopular students, aggressive norms tend to rise, and prosocial norms decline over the school year (Laninga‐Wijnen et al., 2019). Additionally, using cut‐off scores, Laninga‐Wijnen, Harakeh, et al. (2020) classified students into six categories based on prosocial and aggressive behavior and social dominance: socially and nonsocially dominant prosocial, aggressive, and bistrategic adolescents. Classrooms with more socially dominant aggressive and bistrategic students had stronger aggressive norms, while classrooms with a larger presence of socially dominant prosocial students who refrained from aggression were associated with lower aggressive popularity norms. Popularity norms are also associated with bystander behavior in bullying situations. Defending is more likely when bullies are unpopular, as the power balance shifts toward defenders, whereas in classrooms where bullying is rewarded with popularity, defending becomes socially risky (Garandeau et al., 2022; Peets et al., 2015).

In addition to popularity norms, a classroom's social dynamics can also be shaped by other classroom social status norms. In classrooms where bullies are widely disliked—thus being high in *bullying rejection norms*—bystanders find it easier to defend victims (Garandeau et al., 2022), as resisting an unliked bully does not threaten social status. Defenders also fear retaliation less, expecting it will be easier to handle. Similarly, in classrooms with lower *prosocial rejection norms*, where prosocial students are less disliked, students can be expected to be more likely to engage in defending and other prosocial behaviors and less likely to reinforce bullying.

### The current study

The primary aim of the present study is to identify latent profiles of students based on peer‐reported bullying and prosocial behavior and their social status, operationalized as peer‐perceived popularity and likability. Building on Resource Control Theory and previous person‐centered studies, we expect to find distinct profiles, including students characterized by high popularity and bullying behavior, high likability and prosocial behavior, and students exhibiting both bullying and prosocial behaviors (i.e., bistrategic controllers). Existing studies have produced inconsistent findings regarding the existence and characteristics of adolescents who display both aggressive and prosocial behaviors—referred to as *bistrategic controllers*. Some studies have failed to identify this group, while others have found evidence for their distinctiveness in terms of behavior and social status.

Furthermore, relatively little is known about how such profiles relate to students' underlying social motivations and their roles in bullying situations, or how these dynamics unfold within the broader classroom context. To validate these profiles defined by peer‐reported measures, we will examine how they differ in self‐reported bystander behaviors in bullying situations (defending and reinforcing), social goals (popularity and likability goals), and social status insecurity. Based on prior research, we hypothesize that bistrategic students will report the highest levels of both popularity and likability goals, as well as elevated levels of both bullying reinforcement and defending behavior. If confirmed, this pattern would provide additional support for the validity of the bistrategic profile as a socially strategic status‐seeking orientation.

Additionally, given the importance of classroom‐level influences on student behavior and status, this study will adopt a multilevel approach to explore whether classrooms differ in the constellations of individual‐level profiles and how class profiles differ in classroom‐level social status norms, including both bullying and prosocial popularity and rejection norms. As research in this area is still limited, both the identification of classroom‐level profiles and the examination of their associations with social status norms were approached in an exploratory manner, without specific hypotheses. In the present study, the more specific term *bullying popularity/rejection norms*, instead of the broader term *aggressive popularity norms*, is used, as our operationalization was based specifically on peer nominations of bullying behavior (see also Garandeau et al., 2022). Our study thus adopts a narrower perspective that allows for a more precise investigation of how popularity is linked to repeated, intentional harm‐doing within a power‐imbalanced context.

## METHOD

### Participants

Our sample included 6379 participants (50% females; 0.1% students reporting as nonbinary), aged from 12 to 16 (*M*_age_ = 13.43, *SD* = 0.64). Participants were nested in 328 classes in 118 elementary schools. The average classroom size was 21.42 (*SD* = 3.73). Compulsory basic education in Slovenia is organized into a single‐structure (ISCED1 and ISCED2) 9‐year basic school attended by students aged 6 to 15 years. 99% of schools in Slovenia are public schools (Taštanoska, 2022).

Most of the students spoke Slovenian at home (75.3%). Reported ethnicities were 88.5% Slovenian, 5.1% two or more ethnicities (including Slovenian with another ethnicity), 3% Bosnians, and 3.4% all other ethnicities (e.g., Serbian, Albanian, etc.).

### Instruments

#### Peer‐reports

Peer‐reported variables were obtained from peer nominations. For peer‐reported bullying behavior, students were asked who among their classmates is bullying others (i.e., is often being rude to, excluding, or physically abusing some of the classmates). For peer‐reported popularity, students were asked who was the most popular in the classroom. For peer‐reported prosocial behavior, students were asked who is nice to others and is willing to help others. Students were allowed to nominate three classmates, including themselves. Peer‐reported bullying behavior, peer‐reported popularity, and peer‐reported prosocial behavior were calculated for each student and standardized within classrooms (divided by the total possible number of nominators).

Social preference was assessed by using peer nominations regarding whom they liked most and whom they disliked most in their classroom. Participants could provide up to three nominations. A number of nominations received for each student was standardized within classrooms (z‐scores). The social preference score was then calculated by subtracting the “least liked” z‐scores from the “most liked” z‐scores. In addition, the proportion score of “disliked most” nominations received served as the measure of peer rejection used in the computation of classroom‐level rejection norms.

#### Self‐reports

Bystander behavior was measured using Adolescent Peer Relations Instrument: Participant Roles (Parada, 2006) to determine distinct reactions to bullying behavior (i.e., active reinforcer, passive reinforcer, target advocate, and ignore/disregard) with 24 items on a six‐point scale ranging from 1 = not true at all to 6 = completely true. CFA confirmed a good fit of the four‐factor model after some covariances among item errors were added: RMSEA = 0.043, 90% CI [0.042, 0.044], CFI = 0.913; SRMR = 0.056. Only two extreme behaviors were selected for the present study, each consisting of six items: active bullying reinforcement (e.g., “I would join in by calling the student being bullied names”) and defending behavior (e.g., “I would try and protect the student being picked on”). The reliabilities for each subscale were adequate (bullying reinforcement: .72, defending behavior: .89).

Social status goals (i.e., popularity and social preference) and social status insecurity were assessed using a measure developed by Li and Wright (2014). Popularity goals were evaluated with six items (e.g., “I want to be popular among my peers”), social preference goals were assessed with five items (e.g., “I want to be well‐liked by my peers”), and social status insecurity was measured using six items (e.g., “I feel that my social status in the class is threatened.”) on a 5‐point Likert scale, ranging from 1 (never) to 5 (all the time). The reliabilities for each subscale were good (popularity: .84, social preference: .74, social status insecurity: .84). CFA confirmed a good fit of the three‐factor model after some covariances among item errors were added: RMSEA = 0.062, 90% CI [0.060, 0.064], CFI = 0.921; SRMR = 0.067.

#### Classroom norms

On the classroom level, four types of classroom norms were examined: bullying popularity norms, prosocial popularity norms, bullying rejection norms, and prosocial rejection norms. Following the procedure suggested by Dijkstra and Gest (2015), bullying popularity norms were defined as the within‐classroom correlation between peer‐reported bullying behavior and perceived popularity, while prosocial popularity norms were conceptualized as the within‐classroom correlation between peer‐reported prosocial behavior and perceived popularity. Bullying rejection norms were calculated as the within‐classroom correlation between peer‐reported bullying behavior and rejection, while prosocial rejection norms were the within‐classroom correlations between peer‐reported prosocial behavior and rejection. All norms were controlled for the classroom size.

### Procedure

The data were collected as part of a larger longitudinal study with three measurement points aimed at examining the relationship between indicators of classroom social dynamics and students' involvement in bullying during early adolescence. The data used in this study are from the first measurement from the beginning of the school year (October and November 2022). Schools were recruited through a public invitation. All Slovenian elementary schools were invited to take part in the study; based on the schools' responses, ~ 26% of all Slovenian elementary schools were involved. The research was approved by the ethics committee at the Faculty of Arts, University of Maribor. Before the study commenced, school counselors attended a brief online training where they received detailed instructions on conducting the research. In addition, they were provided with comprehensive written guidelines. The study included only 8th‐ and 9th‐grade classrooms where at least 90% of students provided informed consent for their participation both from them and their parents. Students were free to withdraw from the study at any time. The average within‐classroom participation rate was 94.5% (*SD* = 5.4%).

The initial sample comprised 6551 students; however, for the analysis, responses were included from students who completed at least 80% of the items on each questionnaire. The questionnaires were administered in printed form in Slovene, with additional language versions for students with language barriers (i.e., migrant students). All measures were originally adapted into Slovene from established English‐language instruments using a translation and back‐translation procedure following standard guidelines. The Slovene versions have already demonstrated adequate psychometric properties in previous studies with adolescent samples (Košir et al., 2020, 2022). For the present study, additional translations into the most commonly represented migrant languages were prepared by psychology master's students who were native speakers of the respective languages to maximize inclusiveness and participation. These were translations rather than adaptations, created to accommodate the most commonly represented languages in participating classrooms and to maximize student participation. These translated versions were used by a very small proportion of students (0.7% of the total sample). Data were collected through self‐reports and peer nominations. To ensure anonymity, each student was assigned a unique code. A codebook containing all classmates' codes was provided during questionnaire completion, enabling students to use codes instead of names for peer nomination questions. Students had 45 minutes to complete the questionnaire.

### Data analysis

Statistical analyses were performed using IBM Statistics 29.0 and Mplus 8.6. First, missing data analysis revealed that less than 2% of data were missing for the variables of interest. Further, Little's MCAR test was performed, χ^2^(17) = 16.728, *p* = .473, which indicated that data were likely missing at random, which allowed using full information maximum likelihood to handle missing data in Mplus. Since the data were nested, intraclass correlation coefficients were examined (bullying behavior: ICC_school_ = .008, ICC_class_ = .000; prosocial behavior: ICC_school_ = .018, ICC_class_ = .000; popularity: ICC_school_ = .009, ICC_class_ = .000; social preference: ICC_school_ = .000, ICC_class_ = .000; bullying reinforcement: ICC_school_ = .010, ICC_class_ = .031; defending behavior: ICC_school_ = .026, ICC_class_ = .036; popularity goals: ICC_school_ = .011, ICC_class_ = .019; social preference goals: ICC_school_ = .008, ICC_class_ = .030; social status insecurity: ICC_school_ = .003, ICC_class_ = .011; prosocial popularity norms: ICC_school_ = .052; bullying popularity norms: ICC_school_ = .022). Thus, only a small proportion of the total variance was attributable to classroom or school‐level differences. The highest ICCs at the classroom level were observed for prosocial popularity norms, defending behavior, bullying reinforcement, and social preference goals. Although most of the variance was at the individual level, multilevel modeling was conceptually justified given the study's focus on classroom dynamics and peer norms.

### Single and multilevel LPA

First, single‐level LPA was performed in Mplus to determine the number of profiles at the student level based on the proportion scores for bullying behavior, prosocial behavior, popularity, and social preference. Models ranging from one to six profiles were evaluated. Each model estimation was run with 500 random starts and 100 starting iterations, allowing for the replication of the best log‐likelihood value. Once the final number of profiles was determined, the profiles were compared regarding the nominations for bullying behavior, prosocial behavior, popularity, and social preference using Welch's ANOVA (due to unequal variances) in SPSS. After the initial differences between profiles were determined, profiles were compared in terms of gender using Lanza's method (Lanza et al., 2013), in which nonbinary students were not included due to insufficient sample size, and in bystander behavior, social goals, and social status insecurity using the Bolck–Croon–Hagenaars (BCH) approach (Asparouhov & Muthén, 2014) in Mplus.

Second, multilevel LPA was used to examine if, at the classroom level, different profiles of previously identified student‐level profiles emerged (i.e., based on the relative frequency of the student‐level profiles), or put it another way, if there were classrooms with different combinations of student‐level profiles; for example, if there would be classrooms with a higher percent of student‐level profiles that exhibited more bullying behavior. The manual three‐step approach (e.g., Vermunt, 2010) was used to ensure consistency with student‐level profiles, meaning that the profile constraints of the final single‐level profiles (i.e., means and posterior probabilities) were used in the multilevel LPA. The classroom‐level profiles were regressed on student‐level profiles. Multilevel solutions for one to three profiles were tested using several model fit indices. Class profiles were controlled for classroom size, meaning that classroom size was regressed on class profiles. After class profiles were determined, they were compared in prosocial popularity, bullying popularity norms, bullying rejection norms, and prosocial rejection norms using Welch's ANOVA (due to unequal variances) in SPSS.

Model fit for the single‐ and multilevel LPA was evaluated using several indices: Akaike information criterion (AIC), consistent Akaike information criterion (CAIC), Bayesian information criterion (BIC), sample size‐adjusted Bayesian information criterion (SSA‐BIC), and the *p*‐value of the adjusted Lo–Mendell–Rubin likelihood ratio test (pLMR). Lower values for the AIC, CAIC, BIC, and SSA‐BIC indicated better model fit, while for pLMR, a significant *p*‐value suggested that the current profile solution provided a better fit than the previous one. The final solution was selected based on a combination of these indices and theoretical considerations. Although not used as a primary criterion, entropy values are also reported for completeness, with values closer to 1 indicating clearer differentiation between profiles.

## RESULTS

Descriptive statistics and correlations among variables on the student level are reported in Table 1.

**TABLE 1:** Descriptive statistics and correlations of the variables on the student level.

	*M*	*SD*	1.	2.	3.	4.	5.	6.	7.	8.
1. Bullying behavior	0.10	0.17								
2. Popularity	0.11	0.17	.34***							
3. Prosocial behavior	0.12	0.14	−.26***	.16***						
4. Social preference	0.04	1.61	−.20***	.29***	.31***					
5. Bullying reinforcement	1.36	0.63	.27***	.11***	−.15***	−.02				
6. Defending behavior	4.05	1.35	−.12***	−.01	.18***	.03*	−.19***			
7. Popularity goals	2.48	0.86	.12***	.17***	.00	.09***	.19***	−.03**		
8. Social preference goals	3.78	0.83	−.11***	.08***	.19***	.15***	−.09***	.25***	.50***	
9. Social status insecurity	2.27	0.88	−.10***	−.19***	.04***	−.17***	.01	.03*	.26***	.24***

*Note*: Peer‐reported bullying behavior, peer‐reported popularity, and peer‐reported prosocial behavior were calculated for each student and standardized within classrooms (divided by the total possible number of nominators). Social preference is expressed in z‐scores. Ranges for self‐report measures were 1–6 for bystander behaviors (defending and bullying reinforcement) and 1–5 for social goals and social status insecurity. *p < .05, **p < .01, ***p < .001.

### Student‐level profiles

The results of the student‐level LPA are presented in Table 2. With adding another profile, the values of BIC and SSA‐BIC were constantly decreasing. The addition of an extra profile resulted in a consistent reduction in the values of BIC and SSA‐BIC. The elbow plot indicated that the line appeared to flatten around the five‐profile solution. Therefore, profiles with five‐profile and six‐profile solutions were compared to identify the optimal solution, as the final solution should be based on both fit indices and theoretical consideration (Masyn, 2013). Furthermore, each profile should comprise more than 5–8% of the sample (Nylund‐Gibson & Choi, 2018). A comparison of the five‐ and six‐profile solutions demonstrated that the additional profile did not substantially differ from the profile with the highest prosocial behavior. Moreover, the six‐profile solution only over‐extracted the aforementioned profile with the highest prosocial behavior, resulting in adding a profile comprising just 3.3% of the sample.

**TABLE 2:** Fit statistics for single‐level LPA.

	Log‐likelihood	Free parameters	BIC	SSA‐BIC	pLMR	Entropy	Relative frequency of smallest profile
1	−4417.66	8	8905.42	8880.00			
2	−1696.40	13	3506.69	3465.38	<.001	0.969	10.4%
3	−181.16	18	520.02	462.82	<.001	0.938	10.2%
4	682.94	23	−1164.40	−1237.49	<.001	0.938	9%
5	1740.33	28	−3235.36	−3324.33	<.001	0.941	5.3%
6	2372.78	33	−4456.46	−4561.32	<.001	0.924	3.3%
7	2982.68	38	−5632.44	−5753.20	.002	0.931	2.4%
8	3337.04	43	−6297.36	−6434.00	<.001	0.927	2.1%

Abbreviations: BIC, Bayesian information criteria; SSA‐BIC, sample size‐adjusted Bayesian information criteria; *p*LMR, *p*‐value of the adjusted Lo–Mendell–Rubin likelihood ratio test.

**FIGURE 1:**
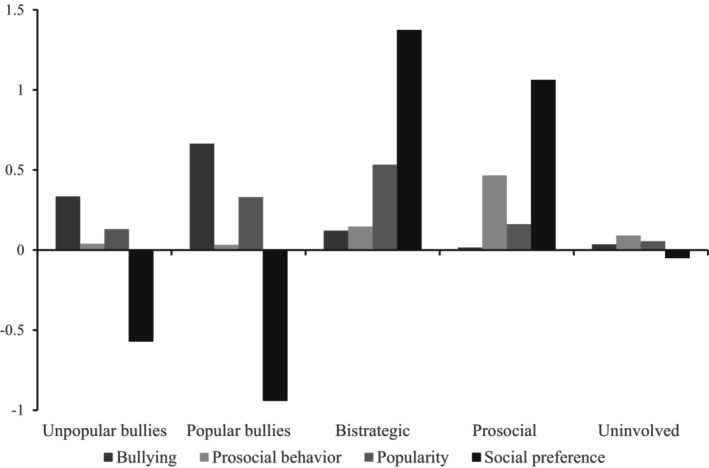
Student‐level LPA profiles.

The most optimal solution was the solution with five profiles (see Figure 1). First, using Welch's ANOVA profiles were compared in bullying behavior, prosocial behavior, popularity, and social preference (see Table A1). Profiles differed in all variables except for Unpopular bullies and Popular bullies profiles, which did not differ in prosocial behavior (*p* = .423).

The first profile, named *Unpopular bullies* profile, comprised 9.6% of the sample. Students in this group were perceived by their peers as high in bullying, less prosocial, unpopular, and having lower social preference. The second profile, referred to as the *Popular bullies* profile, accounted for 5.3% of the sample. This profile was distinguished by the highest levels of bullying behavior, low prosocial behavior, higher popularity, and the lowest social preference among the profiles. The third profile, labeled the *Bistrategic* profile, represented 5.5% of the sample. Students in this profile demonstrated moderate levels of both bullying and prosocial behaviors while having the highest ratings of popularity and social preference. The fourth profile, known as the *Prosocial* profile, included 8.8% of the sample. These students exhibited the lowest levels of bullying behavior, the highest prosocial behavior, relatively low popularity, yet high social preference. Finally, the fifth profile, the *Uninvolved* profile, encompassed the largest proportion of the sample, at 70.7%. Students within this profile were not perceived as bullies, prosocial, or popular, and they had moderate levels of social preference.

After determining the profiles, they were compared in gender using Lanza's method (see Table 3). The overall χ^2^‐test indicated significant differences across profiles, while pairwise comparisons revealed that all profiles significantly differed from each other (all *p*s < .001). Boys were more likely in Unpopular bullies, Popular bullies, and Bistrategic profiles, while girls were more likely to be in Uninvolved and Prosocial profiles.

**TABLE 3:** Profile membership probabilities with standard errors and tests of differences across profiles.

	Boys	Girls
Probabilities (*SE*)		
1. Unpopular bullies	0.89 (0.01)	0.11 (0.01)
2. Popular bullies	0.96 (0.01)	0.04 (0.01)
3. Bistrategic	0.63 (0.03)	0.37 (0.03)
4. Prosocial	0.15 (0.02)	0.85 (0.02)
5. Uninvolved	0.44 (0.01)	0.56 (0.01)
Overall test (Wald χ^2^)^a^	2590.23***	
Pairwise tests (Wald χ^2^)^b^		
1 vs. 2	16.19***	
1 vs. 3	49.49***	
1 vs. 4	1125.51***	
1 vs. 5	749.80***	
2 vs. 3	85.26***	
2 vs. 4	1599.08***	
2 vs. 5	1407.76***	
3 vs. 4	166.41***	
3 vs. 5	244.79***	
4 vs. 5	28.44***	

*Note*: *p < .05, **p < .01, ***p < .001.

^a^ All tests have 2 degrees of freedom.

^b^ All tests have 8 degrees of freedom.

Furthermore, profiles were compared in bystander behavior (i.e., bullying reinforcement, defending) and social goals (i.e., popularity goals, social preference goals, and social status insecurity) using the BCH approach (see Table 4). Regarding bullying reinforcement, students in the Popular bullies profile reported the highest levels of this behavior, followed by those in the Unpopular bullies profile, Bistrategic profile, and Uninvolved profile. The lowest levels of bullying reinforcement were reported in the Prosocial profile. When profiles were compared in defending bullying, the results were reversed: students in the Prosocial profile exhibited the highest levels of defending bullying behavior, followed by those in the Bistrategic profile and the Uninvolved profile (which did not differ from one another). The lowest levels of defending behavior were reported by students in the Popular bullies and Unpopular bullies profiles, which did not differ. Furthermore, profiles were compared in social goals. In particular, students in the Bistrategic profile reported the highest popularity goals among all profiles, followed by those in the Unpopular bullies and the Popular bullies profiles, which did not differ. The lowest levels of popularity goals were reported by students in the Uninvolved and Prosocial profiles, which had no significant differences in popularity goals. Regarding social preference goals, the highest levels of social preference goals were exhibited by students in the Prosocial profile, followed by those in the Bistrategic profile and the Uninvolved profile. Students in the Unpopular bullies and the Popular bullies profiles had the lowest levels of social preference goals. Moreover, a comparison of social status insecurity revealed that the least insecure about their social status were students in the Bistrategic profile, followed by students in the Unpopular bullies and the Popular bullies profiles, who did not differ from one another. The most insecure about their status were students in the Prosocial and Uninvolved profiles (profiles did not differ).

**TABLE 4:** Means and standard deviations of auxiliary variables and tests of mean differences across profiles.

	Bullying reinforcement	Defending behavior	Popularity goals	Social preference goals	Social status insecurity
*M* (*SE*)					
1. Unpopular bullies	1.65 (0.94)	3.68 (1.41)	2.64 (0.97)	3.64 (0.96)	2.15 (0.94)
2. Popular bullies	1.89 (1.08)	3.67 (1.47)	2.75 (1.06)	3.52 (1.01)	2.04 (0.91)
3. Bistrategic	1.40 (0.71)	4.08 (1.44)	2.78 (1.03)	3.96 (0.82)	1.79 (0.77)
4. Prosocial	1.16 (0.36)	4.53 (1.29)	2.46 (0.88)	4.13 (0.69)	2.31 (0.88)
5. Uninvolved	1.30 (0.54)	4.07 (1.41)	2.41 (0.87)	3.76 (0.87)	2.33 (0.94)
Overall test (Wald χ^2^)^a^	1.65 (0.94)	3.68 (1.41)	2.64 (0.97)	3.64 (0.96)	2.15 (0.94)
Pairwise tests (Wald χ^2^)^b^					
1 vs. 2	11.09***	0.02	2.19	2.82	3.28
1 vs. 3	21.37***	16.90***	3.83	28.37***	40.54***
1 vs. 4	140.36***	118.69***	10.89**	103.70***	9.39**
1 vs. 5	77.37***	40.13***	29.97***	8.67***	19.94***
2 vs. 3	48.80***	13.95***	0.14	38.12***	14.32***
2 vs. 4	139.90***	81.60***	16.97***	96.61***	19.87***
2 vs. 5	95.27***	24.00***	31.65***	17.82***	32.73***
3 vs. 4	32.19***	22.73***	21.66***	10.54***	88.20***
3 vs. 5	5.70*	0.02	40.10***	18.12***	153.96***
4 vs. 5	59.82***	62.43***	1.68	134.87***	0.24

*Note*: *p < .05, **p < .01, ***p < .001.

^a^ All tests have 1 degree of freedom.

^b^ All tests have 4 degrees of freedom.

### Classroom‐level profiles

After determining student‐level profiles, classroom‐level LPA was employed to examine if there were different compositions of student‐level profiles among classrooms (i.e., based on the relative frequency of the student‐level profiles). To determine the optimal number of classroom‐level profiles, multilevel solutions with one, two, and three profiles were tested. Classroom‐level profiles were controlled for classroom size. Fit indices for classroom‐level LPA are presented in Table 5. The values of the BIC and SSA‐BIC decreased from one‐ to two‐profile solution, while the BIC slightly increased in the three‐profile solution. After examining the elbow plot, the line also flattened at the two‐profile solution. Thus, the two‐profile solution was selected as the most optimal one.

**TABLE 5:** Fit statistics for classroom‐level LPA.

	Log‐likelihood	Free parameters	BIC	SSA‐BIC	Entropy
1	821.40	10	−1555.20	−1586.98	
2	1827.22	13	−3531.78	−3576.27	0.917
3	1848.74	20	−3522.27	−3585.82	0.900

Abbreviations: BIC, Bayesian information criteria; SSA‐BIC, sample size‐adjusted Bayesian information criteria.

The difference between student‐level profiles within classroom‐level profiles was tested using an omnibus χ^2^‐test of homogeneity, χ^2^(4) = 252.50, *p* < .001. Profile 1 accounted for 80.3% of the classrooms and included a larger proportion of Uninvolved (74%) and a lower proportion of other profiles: Popular bullies (4%), Bistrategic (5%), and Prosocial (7%), while the proportion of Unpopular bullies (10%) was similar to Profile 2. Profile 2 accounted for 19.7% of the classrooms and consisted of a larger proportion of Popular bullies (9%), Bistrategic (9%), and Prosocial (18%) profiles, while the Uninvolved profile was smaller than in Profile 1 (55%). Unpopular bullies profile (10%) was similar to Profile 1. Both classroom‐level profiles were compared in prosocial popularity norms, bullying popularity norms, prosocial rejection norms, and bullying rejection norms. According to the proportion of Uninvolved students in each classroom profile, Profile 1 was named Classrooms with More Uninvolved Students, and Profile 2 was named Classrooms with Fewer Uninvolved Students. Results of Welch's ANOVA revealed that classroom‐level profiles did not differ in prosocial popularity norms (*F*(1, 132.55) = 0.87, *p* = .353, ω^2^ = .003) and bullying popularity norms (*F*(1, 127.73) = 0.21, *p* = .650, ω^2^ = .001). Moreover, profiles differed in prosocial rejection norms (*F*(1, 140.64) = 11.28, *p* = .001, ω^2^ = .031), and bullying rejection norms (*F*(1, 135.08) = 9.04, *p* = .003, ω^2^ = .028). Classrooms with More Uninvolved Students exhibited lower prosocial rejection norms and higher bullying rejection norms than Classrooms with Fewer Uninvolved Students.

## DISCUSSION

The present study aimed to identify distinct profiles of students based on peer‐reported bullying, prosocial behavior, popularity, and likability, with particular interest in whether a bistrategic profile would emerge. Additionally, we examined how these behavior profiles differ in self‐reported bystander behaviors, social status goals, and social insecurity. The study employed a multilevel approach to examine whether classrooms differed in the composition of student behavior status profiles and how these classroom‐level configurations were related to peer norms regarding popularity and rejection of bullying and prosocial behaviors.

### Student‐level findings

Using the student‐level latent profile analysis, five distinct profiles emerged: two with higher bullying behavior, one high in both bullying and prosocial behavior, one with high prosocial behavior, and one average in social preference and low in popularity, bullying, and prosocial behavior. Contrary to our predictions but consistent with some previous findings (Garandeau et al., 2014), two profiles of students high in bullying emerged: Unpopular bullies and Popular bullies. In the Unpopular bullies profile (9.6%), students who exhibited low prosocial behavior and high bullying behavior were found, although their bullying was lower compared to the Popular bullies profile. They were perceived as moderately popular and had low social preference, although slightly higher compared to popular bullies. Students in the Popular bullies profile (5.3%) displayed the highest levels of bullying behavior and low prosocial behavior. As expected, they had high popularity while also scoring the lowest of all profiles on social preference. This is consistent with previous research findings that popular bullies are more likely to be disliked by their peers than students with low levels of bullying behavior (de Bruyn et al., 2010). As hypothesized, the Bistrategic profile (5.5%) showed average levels of both prosocial and bullying behaviors. The students in this profile stood out by having the highest levels of both popularity and social preference. The Prosocial profile (8.8%) was characterized by the highest levels of prosocial behavior and low bullying. They had relatively low popularity but high social preference. The Uninvolved profile scored low in both behavioral dimensions, showing minimal involvement in prosocial and bullying behaviors. Students in this profile had the lowest popularity and low social preference, though higher than both bully profiles. The Uninvolved represented the majority of participants, indicating that only a minority of students were intensely involved in the most prominent social behaviors.

Differences between profiles in bystander behavior, social goals, and social status insecurity were further examined. These constructs have been theoretically and empirically linked to students' motivation for gaining or maintaining social standing (e.g., Caravita & Cillessen, 2012; Ojanen & Findley‐Van Nostrand, 2014) and were therefore expected to provide meaningful differentiation between profiles. In particular, we assumed that a profile truly characterized by bistrategic behavior would not only combine prosocial and aggressive strategies but would also report elevated popularity and likability goals as well as high engagement in both defending and reinforcing behaviors. As such, these comparisons served as an additional validation of the profiles, enabling us to assess whether the patterns captured through peer nominations align with students' own reports of their social motivations and behaviors in bullying contexts.

Both bully profiles reported the highest levels of bullying reinforcement and the lowest defending behavior; since these bystander behaviors can be regarded as subtypes of bullying and prosocial behavior, these findings validate the profiles with self‐reported data. Bullying reinforcement was statistically higher in Popular bullies compared to Unpopular bullies. Consistent with previous findings on the relationships between social motivation and bullying (Caravita & Cillessen, 2012; Košir et al., 2022), both profiles reported the highest popularity goals and the lowest likability goals. Their social status insecurity was low compared to the Uninvolved and Prosocial profiles; however, both bully profiles were more insecure about their social standing than the Bistrategic profile.

The identification of the Bistrategic profile represents a meaningful contribution to the literature, particularly in light of previous inconsistencies regarding its existence (Berger et al., 2015; Hartl et al., 2020; Hawley, 2003). While Berger et al. (2015) did not identify this profile, Hartl et al. (2020) confirmed its existence but did not examine their social preference as a potential additional social resource. Our study not only confirmed the existence of a bistrategic group using peer‐reported behavior and social status but also extended previous findings by showing that these students are both highly popular and well‐liked, indicating that they possess a unique combination of social resources. This is in line with Hawley's (2003) original conceptualization but based on more sophisticated person‐centered statistical techniques.

Students in the Bistrategic profile reported moderate levels of both bullying reinforcement and defending behavior, which is consistent with their relatively high levels of bullying and prosocial behavior. Furthermore, they reported the highest levels of both popularity and likability goals, as well as the lowest social status insecurity. These findings suggest that these students are not only combining prosocial and aggressive behaviors in a strategic manner but are also aware of their social success and are actively motivated to sustain it. Their moderate levels of both bullying reinforcement and defending behavior indicate that they adapt their behavior across different social situations, using both coercive and cooperative strategies when these serve their goals. In this sense, they appear to be highly attuned to the social dynamics of their peer group and capable of adjusting their behavior accordingly. Overall, the bistrategic profile identified in our study confirms all the key hallmarks associated with this group: a dual behavioral strategy (prosocial and aggressive), high social status (popularity and likability), and high social motivation (popularity and likability goals combined with low status insecurity). Thus, while previous findings have been mixed, our results help clarify under what conditions this profile can emerge and how it can be validated through both peer‐ and self‐reports.

Students in the Prosocial profile were characterized by high levels of defending and low levels of bullying, a pattern also identified in previous person‐centered research linking prosocial profiles with defending behavior (Clark et al., 2020). This is consistent with prior findings showing that adolescents who behave prosocially and actively defend victims of bullying tend to be well‐liked by their peers (e.g., Pöyhönen et al., 2010), supporting the notion that defending is a socially valued behavior in early adolescence and closely tied to peer acceptance. In addition, students in the Prosocial profile reported low popularity goals, the highest likability goals, and the highest social status insecurity. This profile thus demonstrated characteristics opposite to both bully profiles. Prosocial students strive for peer acceptance, and given their high social preference, they largely achieve it. Although they reported low popularity aspirations, they were uncertain about their social position within the group. While prosocial students are broadly accepted, they may occupy less socially dominant positions within the peer hierarchy, making their status more contingent on continued norm‐conforming behavior rather than on social power (LaFontana & Cillessen, 2010). Moreover, defending behavior, despite being positively evaluated by peers, can be perceived as socially risky, depending on classroom norms and the relative power of the bully. Previous research suggests that defenders often anticipate potential social costs, even when defending does not objectively lead to rejection (Pöyhönen et al., 2010). Heightened social awareness and moral engagement may therefore contribute to increased concerns about status among prosocial students.

Uninvolved students were the most similar to Prosocial students in terms of bystander behavior, social goals, and social status insecurity. They reported relatively low bullying reinforcement, relatively high defending behavior, low popularity goals, moderate likability goals, and high social status insecurity. This suggests that uninvolved students, as a distinct majority group, play a relatively constructive role in classroom dynamics but are less intensely involved in classroom dynamics than prosocial students, who are similar to them in terms of social motivation and bystander behavior. The major difference between these two groups lies in their likability goals, which are higher in the Prosocial profile—in a subgroup that is more actively engaged in prosocial behavior. This distinction suggests that higher likability goals may motivate more visible prosocial engagement, whereas uninvolved students may endorse similar values but express them in less salient ways. Future research should explore the relationship between likability goals, social preference, and prosocial behavior, as it remains unclear whether likability goals motivate prosocial behavior or if prosocial actions strengthen social preference and likability goals.

Previous studies (e.g., Li & Wright, 2014) reported a weak or nonexistent relationship between social status and social goals, particularly between social preference and likability goals. However, our person‐centered approach‐based findings show that profiles with either high popularity (Popular bullies), high likability (Prosocial), or both (Bistrategic) reported the highest social goals. Uninvolved students, who had relatively low popularity and likability, also reported relatively low social goals. The only profile where a discrepancy between social motivation and status emerged was the Unpopular bullies. These students were not perceived as popular by their peers, yet they reported high popularity goals. Despite this inconsistency, they reported relatively low social status insecurity. According to Resource Control Theory (Hawley, 1999), high aspirations for popularity typically involve a constant self‐produced pressure to defend or enhance one's social status, so one would expect that the discrepancy between their social goals and social standing would lead to high social status insecurity. It is important to note that this group was perceived as less popular by their peers; since previous research (e.g., Peeters et al., 2010) suggests that aggressive students who are unpopular may process social information inaccurately, it might be possible that they overestimated their popularity. The highest levels of social status insecurity were reported by Prosocial and Uninvolved profiles, which are groups with relatively low popularity and high or moderate social preference. These two groups also reported the lowest popularity goals and the highest likability goals. While previous variable‐centered studies have often found that social status insecurity is positively related to popularity goals (e.g., Košir et al., 2022), or that it is highest among adolescents with high popularity and low likability goals (Li & Wright, 2014), our person‐centered findings suggest that social status insecurity may also be present among adolescents who do not actively strive for social status. This aligns with recent work by Lansu and van den Berg (2024), emphasizing the distinction between promotion‐focused and prevention‐focused status motivation—where the latter reflects a desire to avoid low popularity rather than to gain high status. Thus, even students with low explicit status goals may feel vulnerable about their social position, which helps explain the elevated insecurity found in these profiles.

Although gender differences were not the main focus of this study, we examined the likelihood of profile membership by gender. Boys were more likely to be classified into both Bully profiles, and to a lesser extent, into the bistrategic profile. Girls, on the other hand, were more likely to be found in the prosocial profile. No substantial gender differences were observed in the uninvolved profile.

These findings are consistent with previous research, such as Berger et al. (2015), who reported that males were more likely to be aggressive and females more likely to be prosocial. They also partially align with Hawley (2003), who found that boys were overrepresented among coercive controllers, while girls more often fell into prosocial and typical subtypes, with no significant gender differences in the bistrategic group.

### Classroom‐level findings

Additionally, we explored whether these individual‐level profiles differ across classrooms and how distinct classroom‐level configurations—based on the distribution of individual‐level profiles—are associated with classroom norms regarding popularity and rejection of bullying and prosocial behaviors. Using multilevel profile analysis, two distinct classroom‐level profiles emerged: a Profile with More Uninvolved Students (71.6% of participating classrooms) and a Profile with Fewer Uninvolved Students (28.4% of participating classrooms). In Profile with More Uninvolved Students, there were more Uninvolved students and fewer students from Bistrategic, Popular bullies, and Prosocial profiles. In the Profile with Fewer Uninvolved Students, the proportion of Uninvolved students was nearly 20% lower compared to the other profile. This classroom type also included two to three times more students in the Prosocial profile (18% vs. 7%) and nearly twice as many students from Bistrategic and Popular bullies profiles, indicating a higher presence of socially engaged students—both in prosocial and aggressive roles. The two classroom profiles differed in prosocial rejection norms and bullying rejection norms, but not in prosocial or bullying popularity norms. Specifically, classrooms with more Uninvolved students exhibited lower prosocial rejection norms and higher bullying rejection norms compared to classrooms with fewer Uninvolved students. This pattern suggests that in these classrooms, bullying is more clearly sanctioned, while prosocial behavior is less likely to elicit rejection. Although this may seem counterintuitive, our findings suggest that classrooms with more Uninvolved students—that is, classrooms where bullying appears less prevalent or socially salient—may offer more beneficial social environments for students' social learning since in these environments, bullying is more strongly sanctioned, whereas prosocial behavior is more broadly accepted rather than selectively rewarded through popularity. One possible explanation for the more constructive social norms observed in classrooms with a higher proportion of Uninvolved students is that these classrooms may represent more typical or normative classroom ecologies—contexts where bullying is less tolerated and prosocial behavior is socially accepted rather than strongly status‐linked. While Uninvolved students are not highly visible in peer nominations and were less often identified as prosocial or aggressive, they still reported relatively high levels of defending and low reinforcement of bullying. This pattern suggests that, despite their lower social visibility, they may contribute to a classroom climate that discourages bullying. However, we acknowledge that due to the use of a limited peer nomination procedure, the prevalence of Uninvolved students may be overestimated, which complicates any strong claims about their role. An alternative explanation is that the observed classroom profiles primarily reflect differences in the salience of bullying. In this interpretation, classrooms with fewer Uninvolved students and more socially visible profiles (including Popular bullies and Bistrategic students) may simply be classrooms with a relatively higher occurrence of bullying. In contrast, classrooms with more Uninvolved students may be less socially stratified or less dominated by visible behavioral extremes, resulting in weaker associations between bullying and popularity, and clearer rejection of bullying behavior. It is also possible that in classrooms with fewer socially dominant aggressive students, pluralistic ignorance about bullying (Juvonen & Schacter, 2017) may be less prevalent—students might more accurately perceive their peers' disapproval of bullying and thus be less likely to disengage morally. In such environments, even students who are less central in the peer ecology may feel safer acting prosocially or defending victims. This hypothesis should be further investigated in future research.

Neither prosocial nor bullying popularity norms emerged as significant distinguishing factors between classroom profiles. This finding suggests that classroom‐level differences are better captured by rejection‐based norms—that is, by how much bullying or prosocial behaviors are socially disapproved—than by popularity‐based associations. Future research should further explore how these rejection norms shape classroom social climates and whether they interact with popularity norms over time. In future research, it would be valuable to use longitudinal multilevel latent profile analyses to examine the dynamic relationship between classroom popularity norms, other psychosocial classroom characteristics, and students' individual traits.

### Implications, limitations, and further directions

Our research is one of the first to use a multilevel latent profile analysis approach to examine the psychosocial characteristics of classrooms, which is an important advantage given the significant role of classroom social ecology in classroom bullying processes. This method allows us to combine person‐centered analysis approaches with the study of interactive effects between individual and classroom‐level factors in shaping peer group dynamics. Triangulating data sources by identifying profiles based on peer‐reported measures and then examining differences in self‐reported measures reduces the likelihood of common‐method bias and enhances the validity of our findings. Additionally, the focus on Slovenian adolescents offers a unique advantage, as Slovenia's comprehensive, single‐track education system promotes greater within‐classroom heterogeneity and smaller between‐school differences than many WEIRD contexts, providing a valuable setting for examining peer dynamics in socially diverse classrooms.

The emergence of the Bistrategic students profile and the finding that this group of students is not only more popular but also more socially accepted has significant implications for both educational practice and further research. It is likely that this group, with its potentially high influence over peers, maintains a classroom norm that bullying is an acceptable behavior toward certain students. Future research should use social network analyses to further elaborate on the social behavior of this group and explore the hypothesis that they act prosocial toward more influential classmates while participating in bullying against weaker members of the peer group, as suggested by Resource Control Theory (Hawley, 1999). Additionally, this group's potential role should be further considered in programs aimed at fostering an inclusive classroom climate, preventing bullying, and enhancing teachers' capacity for inclusive classroom management and support for social and emotional learning.

It is also important to highlight that some of our findings regarding differences in social status insecurity between profiles are added to previous research, which suggested that social status insecurity may result from a mismatch between social goals and social status (e.g., Košir et al., 2022; Li & Wright, 2014). Our findings do not support this, as the highest social status insecurity was reported by profiles with the lowest popularity aspirations and low popularity. Future research should further explore the reasons for social status insecurity among these groups of students and address the self‐perceptions and experiences of students who have a mismatch between their social aspirations and social standing—this was particularly the case with the Unpopular bullies in our study.

One of the major contributions of our study lies in showing that classroom‐level rejection norms, rather than popularity norms, are significantly related to the composition of students with different psychosocial characteristics. Classrooms where bullying is more clearly disapproved of and prosocial behavior is less likely to elicit rejection tended to include a higher proportion of Uninvolved students and fewer socially visible profiles such as Prosocials, Bistrategic controllers, and Popular bullies. Uninvolved students were most similar to Prosocials in their psychosocial characteristics but were less socially engaged; it is therefore possible that in classrooms with strong bullying rejection norms, visible prosocial behavior may be less necessary to maintain positive peer standing. To foster inclusive classroom relationships and prevent bullying, it is important to strengthen the prosocial behavior of the constructive majority, including defending behavior, as the predominant norm. In this respect, it is relevant to note that the present study is part of a larger research project aimed at developing guidelines that raise students' awareness of their role in bullying. When these guidelines were evaluated in focus groups with early adolescents, they expressed agreement with the guidelines but emphasized that for them to be able to truly follow them, anti‐bullying and prosocial responses need to become the shared classroom norm within the larger peer group (Usenik et al., 2024).

Of course, our study has some limitations. The main limitation is the cross‐sectional research design, which prevents us from drawing conclusions about the direction of relationships between variables. In addition, the study employed a large sample of Slovenian youth. According to the social‐ecological theory of bullying (Espelage & Swearer, 2010), these macro‐level factors may influence students' social behaviors and norms, so the findings should be generalized with caution when considering different cultures. Although relationships between social status measures and other (similar or related) constructs reported in previous studies performed on samples of Slovenian adolescents (Košir et al., 2020, 2022) are similar to those reported in other studies, it is possible that behaviors and the significance of norms may differ between Slovenia and other countries. Specifically, compulsory basic education in Slovenia is organized as a unified, single‐track program, attended by nearly all children in public schools. This structure results in heterogeneous classrooms and relatively small between‐class or between‐school differences, as reflected in the low ICCs in our data. Another limitation concerns the use of limited peer nominations, which may underestimate the visibility or involvement of some students, particularly those categorized as uninvolved, compared to procedures allowing more nominations (Gommans & Cillessen, 2015). Additionally, variations in developmental stages might exist, as early adolescents tend to place more importance on social status compared to childhood and late adolescence. Replicating this study across different cultures and developmental stages could provide deeper insights into the psychosocial subgroups of students in classroom environments with different popularity norms within and across cultures.

## CONCLUSIONS

This study provides significant insights into the social dynamics of early adolescents, identifying five distinct profiles of students based on peer‐reported bullying and prosocial behaviors, popularity, and likability. The most notable contribution is the emergence of the Bistrategic students' profile, who displayed both bullying and prosocial behaviors and were both highly popular and well‐liked. This group also exhibited low social status insecurity, further highlighting their social resourcefulness.

At the classroom level, two distinct classroom profiles emerged. Classrooms with more uninvolved students were characterized by stronger rejection of bullying and weaker rejection of prosocial behavior, but did not differ from other classrooms in popularity norms. These classrooms—with fewer students actively engaging in dominant social behaviors—appeared more favorable for positive social norm structures. Together, these findings emphasize the importance of identifying student and classroom profiles when examining peer dynamics in early adolescence. Recognizing socially influential subgroups—particularly students who behave bistrategically—and understanding classroom‐level patterns linked to clearer disapproval of bullying and greater acceptance of prosocial behavior can inform the development of interventions aimed at promoting inclusive peer relations.

## AUTHOR CONTRIBUTIONS

**Tina Pivec:** Conceptualization; investigation; methodology; visualization; formal analysis. **Katja Košir:** Conceptualization; investigation; funding acquisition; writing – original draft; methodology; project administration; data curation; supervision; resources; writing – review and editing.

## FUNDING INFORMATION

This work was supported by the Slovenian Research Agency (research programs J5‐3114 and P2‐0425). There are no organizations that may gain or lose financially through publication of this manuscript.

## CONFLICT OF INTEREST STATEMENT

The authors report no conflict of interest.

## ETHICS STATEMENT

The study was approved by the local ethics committee at the Faculty of Arts, University of Maribor.

## INFORMED CONSENT

Informed consent was obtained from the parents or legal guardians of all participants, and assent was obtained from the participating adolescents.

## Data Availability

The datasets generated and/or analyzed during the current study are not publicly available but are available from the corresponding author on reasonable request.

## APPENDIX A

**TABLE A1:** Means and standard deviations across student‐level profiles and comparisons between them.

	Unpopular bullies	Popular bullies	Bistrategic	Prosocial	Uninvolved	*F*	ω^2^
Bullying behavior^a^	0.34 (0.09)	0.67 (0.12)	0.12 (0.10)	0.02 (0.04)	0.04 (0.05)	10133.84***	.864
Prosocial behavior^b^	0.04 (0.05)	0.03 (0.05)	0.14 (0.11)	0.48 (0.14)	0.09 (0.09)	2603.92***	.620
Popularity^c^	0.13 (0.14)	0.33 (0.25)	0.54 (0.15)	0.17 (0.17)	0.06 (0.08)	1813.11***	.532
Social preference^d^	−0.57 (1.60)	−0.94 (1.77)	1.37 (1.24)	1.10 (1.20)	−0.05 (1.55)	199.58***	.111

*Note*: Welch's ANOVA was used since the data demonstrated unequal variances. ****p* <  .001.

^a^ *F*(4, 948.94).

^b^ *F*(4, 1050.51).

^c^ F(4, 904.37).

^d^ *F*(3, 1013.64).
